# IP_3_-mediated STIM1 oligomerization requires intact mitochondrial Ca^2+^ uptake

**DOI:** 10.1242/jcs.149807

**Published:** 2014-07-01

**Authors:** Andras T. Deak, Sandra Blass, Muhammad J. Khan, Lukas N. Groschner, Markus Waldeck-Weiermair, Seth Hallström, Wolfgang F. Graier, Roland Malli

**Affiliations:** 1 The Institute of Molecular Biology and Biochemistry, Center of Molecular Medicine, Medical University of Graz, 8010-Graz, Austria; 2 The Institute of Physiological Chemistry, Center of Physiological Medicine, Medical University of Graz, 8010-Graz, Austria

**Keywords:** Mitochondrial Ca^2+^ uptake, UCP2, MCU, SOCE, STIM1 oligomerization

## Abstract

Mitochondria contribute to cell signaling by controlling store-operated Ca^2+^ entry (SOCE). SOCE is activated by Ca^2+^ release from the endoplasmic reticulum (ER), whereupon stromal interacting molecule 1 (STIM1) forms oligomers, redistributes to ER–plasma-membrane junctions and opens plasma membrane Ca^2+^ channels. The mechanisms by which mitochondria interfere with the complex process of SOCE are insufficiently clarified. In this study, we used an shRNA approach to investigate the direct involvement of mitochondrial Ca^2+^ buffering in SOCE. We demonstrate that knockdown of either of two proteins that are essential for mitochondrial Ca^2+^ uptake, the mitochondrial calcium uniporter (MCU) or uncoupling protein 2 (UCP2), results in decelerated STIM1 oligomerization and impaired SOCE following cell stimulation with an inositol-1,4,5-trisphosphate (IP_3_)-generating agonist. Upon artificially augmented cytosolic Ca^2+^ buffering or ER Ca^2+^ depletion by sarcoplasmic or endoplasmic reticulum Ca^2+^-ATPase (SERCA) inhibitors, STIM1 oligomerization did not rely on intact mitochondrial Ca^2+^ uptake. However, MCU-dependent mitochondrial sequestration of Ca^2+^ entering through the SOCE pathway was essential to prevent slow deactivation of SOCE. Our findings show a stimulus-specific contribution of mitochondrial Ca^2+^ uptake to the SOCE machinery, likely through a role in shaping cytosolic Ca^2+^ micro-domains.

## INTRODUCTION

Store-operated Ca^2+^ entry (SOCE) is a common form of Ca^2+^ influx that is linked to important physiological functions of different cell types (Parekh and Putney, 2005). One characteristic feature of SOCE is its activation upon depletion of the endoplasmic reticulum (ER) Ca^2+^ store (Putney, 1986). With the identification of the key molecular constituents of SOCE – the stromal interacting molecule 1 (STIM1) (Zhang et al., 2005; Liou et al., 2005) and the plasma membrane Ca^2+^-pore-forming Orai1 (Vig et al., 2006; Zhang et al., 2006) – the clarification of the elusive molecular mechanism of SOCE has become possible (Soboloff et al., 2012). When the concentration of Ca^2+^ in the ER ([Ca^2+^]_ER_) is reduced, Ca^2+^ dissociates from the luminal EF-hand domain of the ER-membrane-spanning STIM1, initiating its oligomerization (Liou et al., 2007). Subsequently, STIM1 oligomers translocate to subplasmalemmal ER domains, where they form higher-order aggregates, which appear as the so-called ‘STIM1 punctae’ (Park et al., 2009). In this form, STIM1 couples with and activates Orai1 (Park et al., 2009) and other store-operated channels (Cheng et al., 2013), resulting in Ca^2+^ entry. Apart from this function, STIM1 has been shown to regulate the activity of ion pumps (Manjarrés et al., 2010; Ritchie et al., 2012), enzymes (Lefkimmiatis et al., 2009) and cell adhesion proteins (Shinde et al., 2013), pointing to a fundamental role of Ca^2+^-dependent STIM1 oligomerization in cell signaling.

Long before the identification of STIM and Orai proteins and their role in SOCE, mitochondria were shown to contribute to the regulation of SOCE in immune cells (Hoth et al., 1997). Although the exact mechanisms by which mitochondria facilitate SOCE are still unclear, it is assumed that the ability of mitochondria to buffer Ca^2+^ counteracts the Ca^2+^-dependent inactivation of this Ca^2+^-sensitive Ca^2+^ entry pathway (Demaurex et al., 2009; Parekh, 2008b). In addition, mitochondrial Ca^2+^ uptake upon cell stimulation has been suggested to cause a more pronounced depletion of Ca^2+^ from the ER that consequently facilitates SOCE (Demaurex et al., 2009). Because proteins that mediate mitochondrial Ca^2+^ uptake have been identified only recently, the contribution of mitochondrial Ca^2+^ uptake to SOCE has only been investigated indirectly until now. In many studies, mitochondrial Ca^2+^ uptake was diminished by chemical uncouplers, such as carbonyl cyanide-4-(trifluoromethoxy)phenylhydrazone (FCCP) or antimycin A, an inhibitor of complex III of the respiratory chain, which resulted in a significant reduction in SOCE (Naghdi et al., 2010; Hoth et al., 1997; Glitsch et al., 2002). In line with these findings, energized mitochondria were shown to result in increased SOCE (Gilabert and Parekh, 2000). Although these findings point to the importance of mitochondria in SOCE regulation, the actual role of mitochondrial Ca^2+^ uptake in the process of SOCE activation and maintenance under physiological conditions remains elusive.

In this study, mitochondrial Ca^2+^ uptake in HeLa cells was strongly diminished by a stable knockdown of either MCU (Baughman et al., 2011; De Stefani et al., 2011) (MCU^KD^) or UCP2 (Trenker et al., 2007; Trenker et al., 2008) (UCP2^KD^), two proteins of the inner mitochondrial membrane, which have been shown to be involved in mitochondrial Ca^2+^ uptake (Waldeck-Weiermair et al., 2013). MCU, the proposed core component of a ubiquitous mitochondrial Ca^2+^ channel, contributes to mitochondrial Ca^2+^ uptake regardless of the source and mode of Ca^2+^ mobilization (Baughman et al., 2011; De Stefani et al., 2011), whereas UCP2 has been shown to mediate principally the uptake of Ca^2+^ from areas of high Ca^2+^ concentration – Ca^2+^ micro-domains, which are formed upon ER Ca^2+^ release (Waldeck-Weiermair et al., 2013). Accordingly, the individual knockdown of these proteins enabled us to distinguish whether mitochondrial uptake of intracellularly released Ca^2+^ or of Ca^2+^ entering the cell is determinant for SOCE. Our data demonstrate that UCP2- and MCU-dependent mitochondrial uptake of inositol-1,4,5-trisphosphate (IP_3_)-dependent intracellularly released Ca^2+^ represents an essential step in the activation of STIM1 and, hence, SOCE. Correlations between the dynamics of ER Ca^2+^ depletion and STIM1 oligomerization upon different modes of Ca^2+^ mobilization suggest that mitochondrial Ca^2+^ sequestration predominately shapes IP_3_-mediated cytosolic Ca^2+^ micro-domains, which facilitates STIM1 oligomerization under physiological conditions of cell stimulation. In addition, MCU-dependent mitochondrial buffering of entering Ca^2+^ is crucial for the maintenance of SERCA-inhibition-induced SOCE signals. In summary, we highlight a special and tight regulation of STIM1 activation and SOCE maintenance by mitochondrial Ca^2+^ uptake upon physiological and non-physiological stimuli.

## RESULTS

### Stable knock-down of either MCU or UCP2 inhibits mitochondrial Ca^2+^ uptake and impairs STIM1 oligomerization upon IP_3_-mediated Ca^2+^ release

In line with recent reports (Baughman et al., 2011; De Stefani et al., 2011; Waldeck-Weiermair et al., 2013) a stable knockdown of MCU in HeLa cells (supplementary material Fig. S1A) strongly reduced mitochondrial sequestration of intracellularly released (supplementary material Fig. S1B) and entering Ca^2+^ (supplementary material Fig. S1C). By contrast, HeLa cells stably depleted of UCP2 (supplementary material Fig. S1A) showed an impaired mitochondrial Ca^2+^ signal upon intracellular Ca^2+^ mobilization, whereas mitochondrial uptake of entering Ca^2+^ was not affected (supplementary material Fig. S1B,C). These results confirm our recent findings, which have revealed the exclusive involvement of UCP2 and UCP3 in mitochondrial Ca^2+^ sequestration upon IP_3_-mediated intracellular Ca^2+^ release (Waldeck-Weiermair et al., 2011; Trenker et al., 2007; Waldeck-Weiermair et al., 2010b). As mitochondria play a central role in cellular energy homeostasis (Graier et al., 2007; Duchen, 2004), we tested whether stable silencing of either MCU or UCP2 altered the basal metabolic status of HeLa cells. For this purpose, we measured the mitochondrial membrane potential (supplementary material Fig. S1D), oxygen consumption rate (supplementary material Fig. S1E) and cellular ATP content (supplementary material Fig. S1F) in stable knockdown cells. All of these variables remained unaffected by the knockdown of MCU or UCP2. Moreover, the morphology of mitochondria (supplementary material Fig. S1G,H), ER structure (supplementary material Fig. S1G) and the contact sites between these organelles (supplementary material Fig. S1I) were not altered by the stable knockdown of MCU or UCP2. In summary, these data demonstrate that the knockdown of MCU or UCP2 results in specific effects on distinct modes of mitochondrial Ca^2+^ uptake. Therefore, MCU^KD^ and UCP2^KD^ cells can serve as suitable models to specifically investigate the role of mitochondrial Ca^2+^ sequestration in SOCE regulation. In addition, due to the selective engagement of UCP2 in mitochondrial Ca^2+^ sequestration depending on the source of Ca^2+^ (supplementary material Fig. S1B,C) (Waldeck-Weiermair et al., 2010b</emph>; Waldeck-Weiermair et al., 2011), these cell models allowed us to separately examine whether mitochondrial buffering of intracellularly released or entering Ca^2+^ contributes to SOCE activation.

**Fig. 1.**
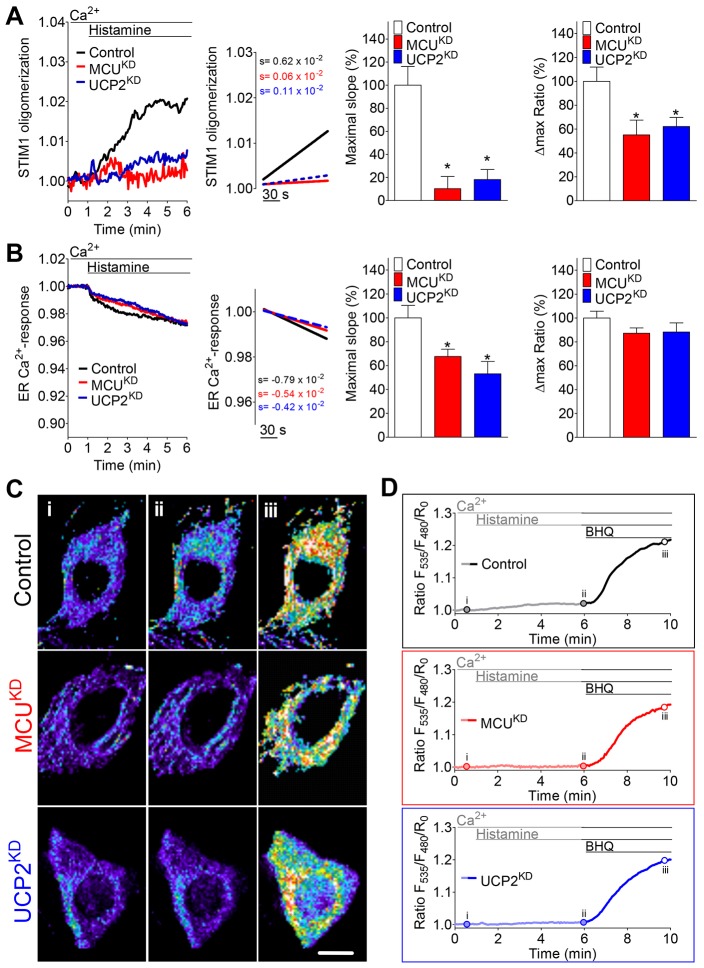
**IP_3_-mediated STIM1 oligomerization is impaired in MCU^KD^ and UCP2^KD^ cells.** (A,B) Left-most panels, dynamic changes in the ratio of F_535_/F_480_ [indicating STIM1 oligomerization (A) or ER Ca^2+^-response (B)] over time, normalized to the basal ratio (R_0_). Middle-left panels; Curves were fitted with straight lines using linear regression to assess the maximal slope (s) of the increase (A) or decrease (B) of the ratio F_535_/F_480_/R_0_. Scale bar indicates 30 seconds (30 s). Middle-right panels; data show the mean±s.e.m. of the maximal slope normalized to control (set at 100%). Right-most panels; data show the mean±s.e.m. of the Δmax ratio F_535_/F_480_/R_0_ normalized to control. *P<0.05 (unpaired Student's *t*-test). (A) STIM1 oligomerization induced by 100 µM histamine in the presence of extracellular Ca^2+^ was visualized in control (black, *n* = 35), MCU^KD^ (red, *n* = 37) and UCP2^KD^ (blue, *n* = 33) cells by measuring intermolecular FRET between CFP–STIM1 and YFP–STIM. (B) Dynamics of ER Ca^2+^ depletion (ER Ca^2+^-response) were monitored in control, MCU^KD^ and UCP^KD^ (*n* = 33, 33 and 35, respectively) cells expressing D1ER in the same experimental protocol described in A. (C) Respective pseudocolor CFP/YFP FRET images of control, MCU^KD^ and UCP2^KD^ cells under basal conditions (i), after 100 µM histamine (ii) and after addition of 15 µM BHQ (iii). Increased FRET signals appear as red pixels. Scale bar: 10 µm. (D) Time-lapse FRET measurement of STIM1 oligomerization in the control, MCU^KD^ and UCP2^KD^ cells shown in B. The thin regions of the curves (0–6 minutes) are identical to those shown in the left-most panel of A, without *y*-axis magnification.

The initial step of SOCE activation is accomplished by the formation of STIM1 oligomers upon reduction of [Ca^2+^]_ER_ (Luik et al., 2008). By measuring Förster resonance energy transfer (FRET) between STIM1 proteins fused to either cyan or yellow fluorescent proteins (CFP or YFP, respectively), the dynamic process of STIM1 oligomerization can be visualized and quantified (Liou et al., 2007; Deak et al., 2013). We used this approach to investigate whether impaired mitochondrial Ca^2+^ uptake induced by the stable knockdown of MCU or UCP2 affects STIM1 oligomerization upon ER Ca^2+^ mobilization under physiological conditions (Fig. 1). Cell treatment with histamine in the presence of extracellular Ca^2+^ resulted in a moderate, but significant increase in the FRET signal between CFP–STIM1 and YFP–STIM1 in control cells (Fig. 1A). However, this increase was almost absent in MCU^KD^ and UCP2^KD^ cells (Fig. 1A). The measurement of [Ca^2+^]_ER_ in response to histamine using D1ER, an ER-targeted genetically encoded Ca^2+^ probe (Palmer et al., 2004), revealed that the reduction in the global ER Ca^2+^ concentration was slightly slower in the knockdown cells compared with that of control cells (Fig. 1B). The subsequent addition of the SERCA inhibitor 2,5-di-t-butyl-1,4-benzohydroquinone (BHQ), which causes a strong depletion of the ER Ca^2+^ store, considerably augmented STIM1 oligomerization in all three cell types (Fig. 1C,D). These findings indicate that, upon IP_3_-mediated depletion of ER Ca^2+^, the subsequent oligomerization of STIM1 proteins is facilitated by MCU- and UCP2-dependent mitochondrial Ca^2+^ uptake. Notably, after ∼4 minutes of histamine treatment, the degree of ER Ca^2+^ depletion in MCU^KD^ and UCP2^KD^ cells reached that of control cells (Fig. 1B), whereas the STIM1 FRET signals in the knock-down cells remained strongly reduced (Fig. 1A). This suggests that mitochondrial Ca^2+^ sequestration does not just contribute to STIM1 activation by shaping the ER Ca^2+^ response.

### The contribution of mitochondrial Ca^2+^ uptake to STIM1 oligomerization is determined by the mode of Ca^2+^ mobilization

**Fig. 2.**
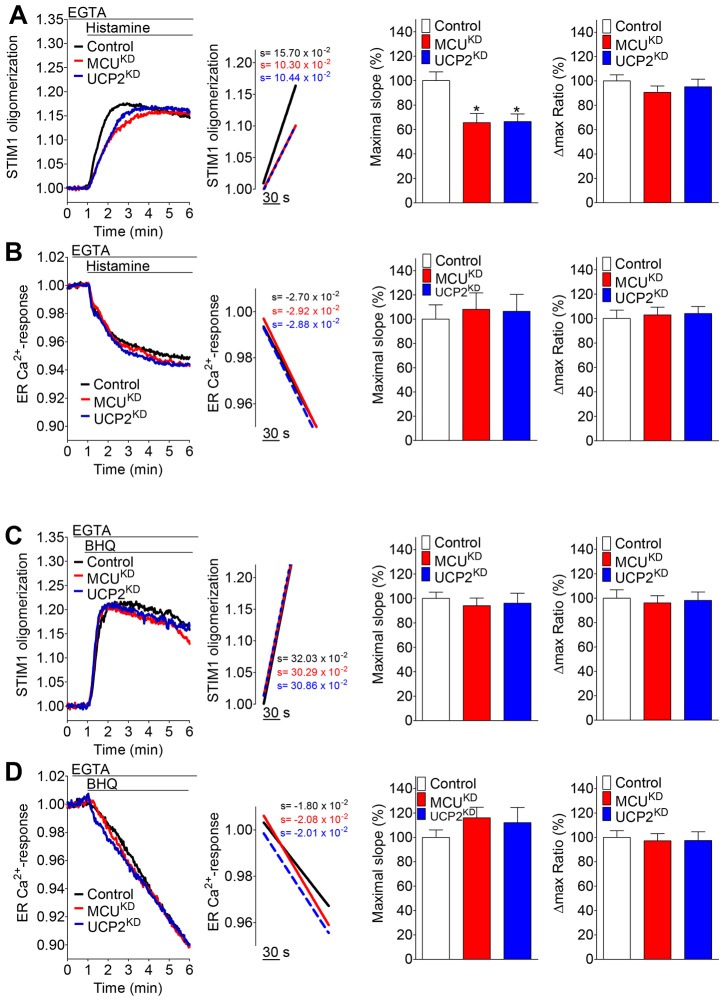
**Stimulus-specific dependency of STIM1 oligomerization on mitochondrial Ca^2+^ uptake.** Left-most panels, dynamic changes of the ratio F_535_/F_480_ [indicating STIM1 oligomerization (A,C) or ER Ca^2+^ response (B,D)] over time normalized to the basal ratio (R_0_). Middle-left panels, curves were fitted with straight lines using linear regression to assess the maximal slope (s) of the increase (A,C) or decrease (B,D) in the ratio F_535_/F_480_/R_0_. Scale bar indicates 30 seconds (30 s). Middle-right panels, data show the mean±s.e.m. of the maximal slope normalized to control (set at 100%). Right-most panels, data show the mean±s.e.m. of the Δmax ratio F_535_/F_480_/R_0_ normalized to control. (A) Control, MCU^KD^ and UCP2^KD^ cells (*n* = 65, 55 and 55, respectively) coexpressing YFP–STIM1 and CFP–STIM1 were stimulated with 100 µM histamine in the absence of extracellular Ca^2+^ (EGTA). *P<0.05 compared with control (unpaired Student's *t*-test). (B) The ER Ca^2+^ response of control, MCU^KD^ and UCP2^KD^ cells (*n* = 31, 36 and 27, respectively) expressing D1ER in the same experimental protocol as in A. (C) Control, MCU^KD^ and UCP2^KD^ cells (*n* = 22, 24 and 22, respectively) coexpressing YFP–STIM1 and CFP–STIM1 were stimulated with 15 µM BHQ in the absence of extracellular Ca^2+^ (EGTA). (D) The ER Ca^2+^ response of control, MCU^KD^ and UCP2^KD^ cells (*n* = 35, 45 and 38, respectively) expressing D1ER in the same experimental protocol as in C.

To further characterize the role of mitochondrial Ca^2+^ buffering in IP_3_-mediated STIM1 activation and ER Ca^2+^ depletion, cells were stimulated with histamine in the absence of extracellular Ca^2+^. This led to an enhanced and rapid STIM1 oligomerization (Fig. 2A) due to a pronounced release of Ca^2+^ from the ER (Fig. 2B). Although the ER depleted at the same rate in all three cell lines under these conditions (Fig. 2B), STIM1 oligomerization was clearly delayed in MCU^KD^ and UCP2^KD^ cells compared with that of the control cells (Fig. 2A). However, neither STIM1 oligomerization (Fig. 2C) nor ER Ca^2+^ release (Fig. 2D) was affected by MCU or UCP2 knockdown when ER Ca^2+^ was mobilized by the SERCA inhibitor BHQ. These findings show a stimulus-specific contribution of mitochondrial Ca^2+^ buffering to STIM1 oligomerization dynamics. Moreover, ER Ca^2+^ responses were insensitive to MCU or UCP2 silencing under these conditions (Fig. 2B,D), confirming that mitochondrial Ca^2+^ uptake promotes STIM1 activation by a mechanism other than the control of ER Ca^2+^-responses only.

### Increasing cytosolic Ca^2+^ buffering with BAPTA diminishes the contribution of mitochondrial Ca^2+^ uptake to IP_3_-induced STIM1 oligomerization

**Fig. 3.**
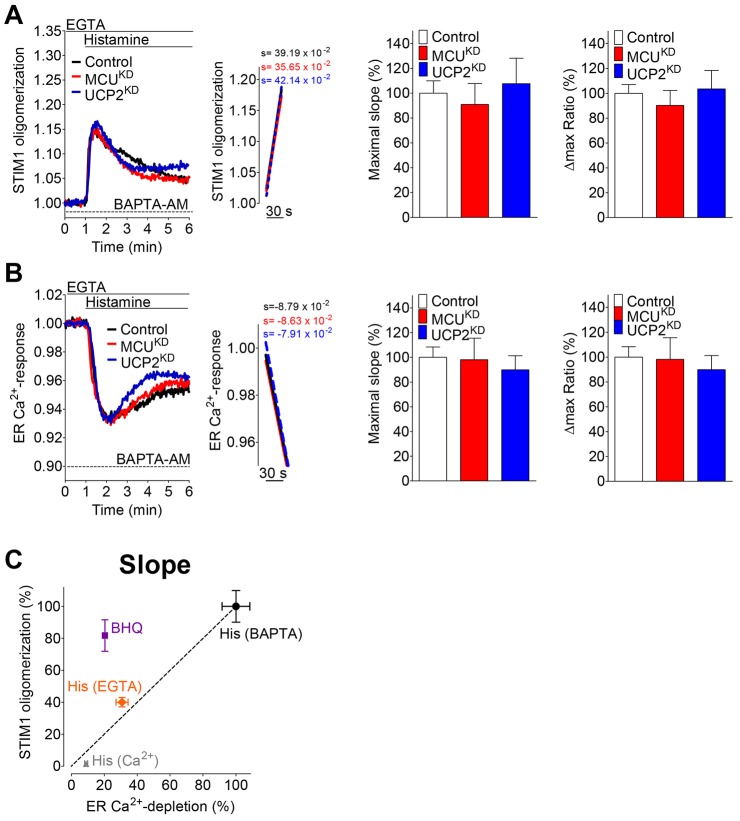
**IP_3_-mediated STIM1 oligomerization does not require intact mitochondrial Ca^2+^ uptake in BAPTA-AM-loaded cells.** (A,B) Left-most panels, dynamic changes in the ratio F_535_/F_480_ [indicating STIM1 oligomerization (A) or ER Ca^2+^ response (B)] over time normalized to the basal ratio (R_0_). Middle-left panels, curves were fitted with straight lines using linear regression to assess the maximal slope (s) of the increase (A) or decrease (B) of the ratio F_535_/F_480_/R_0_. Scale bar indicates 30 seconds (30 s). Middle-right panels, data show the mean±s.e.m. of the maximal slope normalized to control (set at 100%). Right-most panels, data show the mean±s.e.m. of the Δmax ratio F_535_/F_480_/R_0_ normalized to control. (A) Prior to experiments, control, MCU^KD^ and UCP2^KD^ cells (*n* = 16, 10 and 12, respectively) coexpressing YFP–STIM1 and CFP–STIM1 were exposed to 10 µM BAPTA-AM for 45 minutes and were stimulated with 100 µM histamine in a Ca^2+^-free solution (EGTA). (B) The ER Ca^2+^ response of control, MCU^KD^ and UCP2^KD^ cells (*n* = 16, 15 and 19, respectively) expressing D1ER in the same experimental protocol as in A. (C) Initial slopes of ER Ca^2+^ depletion (*x*-axis) were plotted against maximal slopes of STIM1 oligomerization (*y*-axis) of control cells that were treated with 100 µM histamine in the presence of 2 mM Ca^2+^ (grey triangle; *n* = 35 for STIM1 oligomerization, *n* = 33 for ER Ca^2+^ response), 100 µM histamine in EGTA (orange diamond; *n* = 65 for STIM1 oligomerization, *n* = 31 for ER Ca^2+^ depletion), 15 µM BHQ in EGTA (purple square; *n* = 22 for STIM1 oligomerization, *n* = 35 for ER Ca^2+^ response) and 100 µM histamine in BAPTA-AM-loaded cells (black circle; *n* = 16 for each). Data represent the mean±s.e.m., normalized to the maximal slopes in BAPTA-AM-loaded cells, which were defined as 100%.

Global and local cytosolic Ca^2+^ elevations were shown to impair STIM1 activation independently of [Ca^2+^]_ER_ (Malli et al., 2008; Shen et al., 2011b). Accordingly, upon IP_3_-mediated release of Ca^2+^ from the ER, mitochondrial buffering of cytosolic Ca^2+^ might counteract this negative feedback and, thus, facilitate STIM1 activation. In order to experimentally prove this hypothesis, we investigated histamine-induced STIM1 oligomerization and ER Ca^2+^ release in cells loaded with the chemical Ca^2+^ chelator 1,2-bis(2-aminophenoxy)ethane-N,N,N′,N′-tetraacetic acid tetrakis(acetoxymethyl ester) (BAPTA-AM), which accumulates in the cytosol and prevents the formation of high-concentration cytosolic Ca^2+^ micro-domains (Parekh, 2008a). In cells loaded with BAPTA-AM, STIM1 oligomerization (Fig. 3A) and ER Ca^2+^ depletion (Fig. 3B) in response to histamine were rapid and occurred identically in MCU^KD^, UCP2^KD^ and control cells. However, both STIM1 oligomerization and ER Ca^2+^ depletion were transient in BAPTA-AM-loaded cells during treatment with histamine (Fig. 3). The transient nature of both signals most likely reflects SERCA-dependent replenishment of ER Ca^2+^ from the BAPTA-chelated cytosolic Ca^2+^. Nevertheless, BAPTA-AM loading abolished the contribution of mitochondrial Ca^2+^ uptake to histamine-induced STIM1 oligomerization. This points to a possible role for mitochondria in shaping high-concentration cytosolic Ca^2+^ micro-domains that negatively regulate STIM1 activation upon IP_3_-mediated Ca^2+^ mobilization independently of the ER Ca^2+^ content. A relative independency of STIM1 activation from the speed of ER Ca^2+^ depletion becomes obvious by correlating the BHQ-induced initial maximal slope of STIM1 oligomerization with the ER Ca^2+^ depletion kinetics (Fig. 3C). Although the SERCA inhibitor evoked a slow ER Ca^2+^ depletion (Fig. 2D; Fig. 3C), the kinetics of STIM1 oligomerization were quite fast (Fig. 2C; Fig. 3C). This mismatch further points to a role of cytosolic Ca^2+^ in the regulation of STIM1 activation.

### SOCE activity upon stimulation with an IP_3_-generating agonist is attenuated in cells depleted of MCU or UCP2

**Fig. 4.**
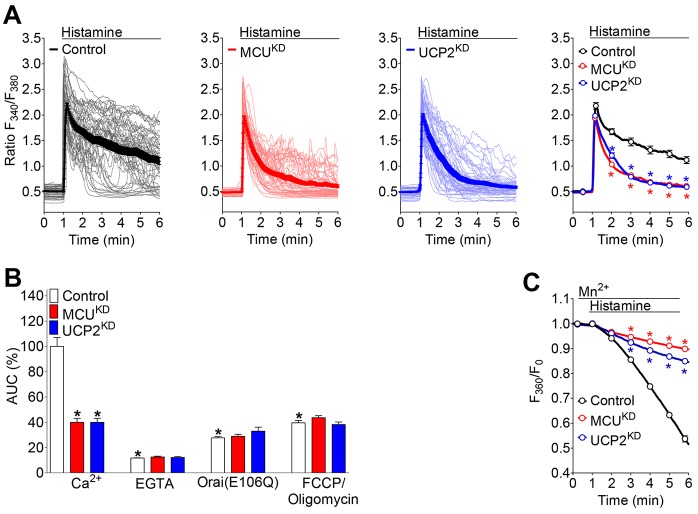
**IP_3_-triggered Ca^2+^ entry is attenuated in MCU^KD^ and UCP2^KD^ cells.** (A) Curves indicate single-cell cytosolic Ca^2+^ signals (thin lines) and their respective mean±s.e.m. (thick lines) upon addition of 100 µM histamine to fura-2/am-loaded control cells (left panel, *n* = 51), MCU^KD^ cells (middle-left panel, *n* = 57) and UCP2^KD^ cells (middle-right panel, *n* = 60). Right-most panel, data show the mean±s.e.m. of the Ca^2+^ responses of control (black), MCU^KD^ (red) and UCP^KD^ (blue) cells. (B) The area under the curve (AUC) corresponding to the respective cytosolic Ca^2+^ responses shown in A and in supplementary material Fig. S2 was calculated for each sample and normalized to the control cytosolic Ca^2+^ responses in the presence of 2 mM Ca^2+^ in the extracellular medium (set at 100%). The data show the mean±s.e.m. The number of control, MCU^KD^ and UCP2^KD^ cells for each condition are as follows; Ca^2+^ (*n* = 51, 57 and 60, respectively), EGTA (*n* = 76, 76 and 65, respectively), Orai(E106Q) (*n* = 43,39 and 36, respectively), FCCP/Oligomycin (*n* = 54, 40 and 56, respectively). (C) Perfusion with 100 µM Mn^2+^ quenched cytosolic fura-2 fluorescence in control, MCU^KD^ and UCP2^KD^ cells (*n* = 43, 56 and 52, respectively) upon treatment with 100 µM histamine. Data show the mean±s.e.m. of normalized fluorescence intensities measured at 360 nm excitation (F_360_/F_0_); *P<0.05 versus control (unpaired Student's *t*-test).

The impact of decelerated IP_3_-mediated STIM1 activation on Ca^2+^ entry was examined using the fura-2 technique in MCU^KD^ and UCP^KD^ cells. In wild-type cells, treatment with histamine in the presence of extracellular Ca^2+^ generated sustained cytosolic Ca^2+^ signals (Fig. 4A), whereas, in the absence of extracellular Ca^2+^, histamine-evoked Ca^2+^ signals became transient (Fig. 4B; supplementary material Fig. S2A); thus indicating that Ca^2+^ entry is required to maintain elevated cytosolic Ca^2+^ levels during stimulation. By contrast, in MCU- or UCP2-depleted cells, the SOCE-dependent second phase of cytosolic Ca^2+^ elevation was strongly reduced (Fig. 4A).

To verify whether the Ca^2+^ influx induced by IP_3_-mediated ER depletion is indeed accomplished by the STIM1–Orai1-driven SOCE pathway, HeLa cells were transfected with Orai1(E106Q), a dominant-negative form of the wild-type Orai1 protein (Vig et al., 2006). Ca^2+^ responses upon stimulation of control cells expressing Orai1(E106Q) showed a strongly reduced plateau phase in the presence of extracellular Ca^2+^ (Fig. 4B; supplementary material Fig. S2B). Notably, the already transient elevation of cytosolic Ca^2+^ in response to histamine in the MCU^KD^ and UCP2^KD^ cells remained unaffected by an additional expression of the dominant-negative Orai (Fig. 4B; supplementary material Fig. S2B). This indicates that STIM1–Orai1-dependent SOCE is not activated in cells lacking the mitochondrial Ca^2+^ sequestration of intracellularly released Ca^2+^. Similar results were obtained in all cell models (i.e. wild-type, MCU^KD^ and UCP2^KD^ cells) upon incubation with FCCP and oligomycin A prior to histamine stimulation (Fig. 4B; supplementary material Fig. S2C). Interestingly, the sustained phase of the cytosolic Ca^2+^ signal in MCU- and UCP2-silenced cells in the absence of FCCP and oligomycin A was reduced to the same level as in control cells pre-exposed to the drugs (Fig. 4B), indicating that mitochondrial Ca^2+^ uptake is the molecular mechanism by which mitochondria contribute to SOCE under these conditions. To additionally confirm the reduced SOCE in MCU^KD^ and UCP2^KD^ cells, the rate of Mn^2+^ influx was measured (Fig. 4C). Cytosolic fura-2 fluorescence in response to histamine was quenched at a significantly slower rate by Mn^2+^ in MCU^KD^ and UCP2^KD^ compared with controls cells (Fig. 4C), indicating that SOCE was indeed reduced in cells lacking mitochondrial Ca^2+^ uptake.

### Re-expression of MCU restores both IP_3_-mediated mitochondrial Ca^2+^ uptake and SOCE

**Fig. 5.**
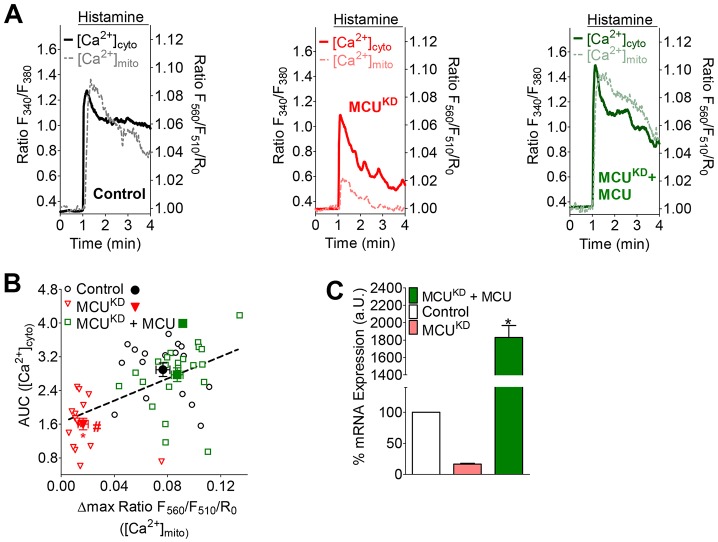
**Re-expression of MCU in MCU^KD^ cells restores both IP_3_-mediated mitochondrial Ca^2+^ uptake and SOCE.** (A) Representative cytosolic (thick lines) and mitochondrial (thin lines) Ca^2+^ signals from the same individual 4mtD1GO-expressing and fura-2/am-loaded control cells (left-most panel, black), MCU^KD^ cells (middle panel, red) and MCU-transfected MCU^KD^ cells (right panel, green). (B) Scatter plot correlating cytosolic and mitochondrial Ca^2+^ responses (black dotted line) shown in A. The values of the area under the curve (AUC) of cytosolic Ca^2+^ signals (arbitrary units; *y*-axis) were plotted against peak mitochondrial Ca^2+^ uptake (Δmax Ratio F_560_/F_510_/R_0_; *x*-axis) derived from the same individual control (open black symbols, *n* = 17), MCU^KD^ (open red symbols, *n* = 17) and MCU^KD^ + MCU (open green symbols, *n* = 22) cells. Filled symbols represent the mean±s.e.m; *P<0.05 versus control; ^#^*P*<0.05 versus control (unpaired Student's *t*-test). (C) Relative mRNA levels (arbitrary units, AU) of MCU in control cells (*n* = 9) and MCU^KD^ cells (*n* = 9), as shown in supplementary material Fig. S1A, as well as MCU^KD^ cells expressing MCU (*n* = 9). Data show the mean±s.e.m; *P<0.05 versus control.

To correlate histamine-evoked cytosolic Ca^2+^ signals with changes in [Ca^2+^]_mito_ on the single-cell level, Ca^2+^ responses in both compartments were measured simultaneously using a red-shifted mitochondrially targeted cameleon (a protein that allows the visualization of Ca^2+^ levels in live cells) in combination with fura-2 (Waldeck-Weiermair et al., 2012). This approach revealed a positive correlation between mitochondrial Ca^2+^ uptake and the plateau phase of [Ca^2+^]_cyto_ in response to histamine (Fig. 5A,B). Notably, due to oscillating cytosolic Ca^2+^ responses, the correlation was rather weak. However, a transient re-expression of MCU in MCU^KD^ cells efficiently rescued the mRNA levels of MCU (Fig. 5C) and restored both mitochondrial Ca^2+^ uptake and the plateau phase of the cytosolic Ca^2+^ signal in the same individual cells (Fig. 5A,B). Thus, these results confirm that mitochondrial Ca^2+^ uptake contributes to SOCE upon cell stimulation with histamine.

### MCU-dependent mitochondrial buffering of entering Ca^2+^ is essential for the maintenance of thapsigargin-induced SOCE

**Fig. 6.**
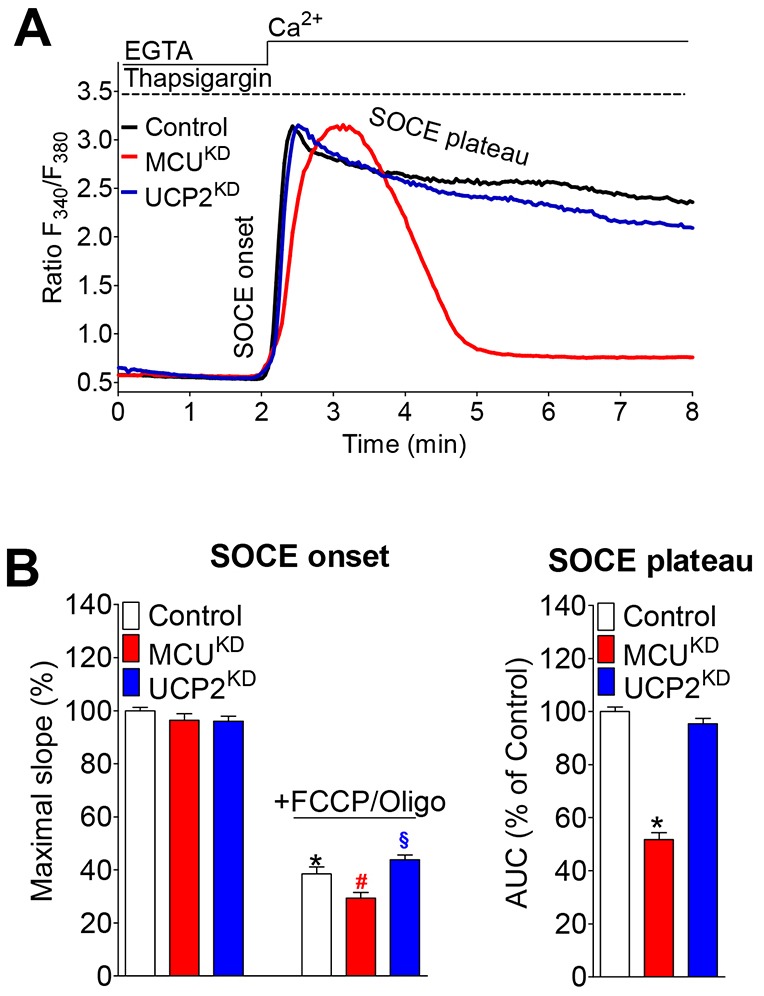
**The maintenance of thapsigargin-induced SOCE relies on MCU-dependent mitochondrial Ca^2+^ buffering.** (A) Curves represent cytosolic Ca^2+^ responses of control, MCU^KD^ and UCP2^KD^ cells upon addition of 2 mM Ca^2+^. Cells were pre-stimulated with 1 µM thapsigargin to induce depletion of the ER Ca^2+^ store. (B) Left panel, curves were fitted with straight lines using linear regression to assess the maximal slope of the increase in [Ca^2+^]_cyto_ (SOCE onset) upon Ca^2+^ addition. As a negative control, cells were exposed to FCCP and oligomycin (2 µM each) prior to the addition of Ca^2+^. The number of control, MCU^KD^ and UCP2^KD^ cells for each condition are: untreated, *n* = 104, 93 and 91, respectively; +FCCP/oligomycin, *n* = 90, 77 and 99, respectively. Right panel, Values of the area under the curve (AUC) of SOCE-driven Ca^2+^ signals were calculated to quantify inactivation (SOCE plateau). Data show the mean±s.e.m. normalized to control (set at 100%); *P<0.05 versus control; ^#^*P*<0.05 versus MCU^KD^; ^§^*P*<0.05 versus UCP2^KD^.

A classical protocol to image SOCE is based on a complete ER Ca^2+^ depletion with a SERCA inhibitor in the absence of extracellular Ca^2+^ and the subsequent addition of Ca^2+^, which leads to a pronounced, SOCE-mediated elevation of [Ca^2+^]_cyto_ within these pre-stimulated cells (Parekh and Putney, 2005). The initial phase of this cytosolic SOCE signal was affected in neither MCU^KD^ nor UCP2^KD^ cells (Fig. 6A), which is in line with the unaltered STIM1 oligomerization triggered by SERCA inhibition (Fig. 2C). By contrast, incubating the cells with FCCP and oligomycin A prior to the addition of Ca^2+^ in the same experimental protocol suppressed cytosolic Ca^2+^ elevations to the same level in all three cell types (Fig. 6B), indicating that SOCE triggered by SERCA inhibition requires intact mitochondria, but not mitochondrial Ca^2+^ buffering, for activation. However, exclusively in MCU^KD^ cells, which show reduced mitochondrial accumulation of entering Ca^2+^ (supplementary material Fig. S1C), the thapsigargin-induced SOCE signal was almost completely terminated within several minutes (Fig. 6A,B). This finding suggests that intact MCU-dependent mitochondrial buffering of entering Ca^2+^ is required for the maintenance of SOCE, even if this Ca^2+^ entry pathway is maximally activated by irreversible SERCA inhibition.

## DISCUSSION

In this study, the significance of mitochondrial Ca^2+^ uptake in SOCE activation and maintenance in HeLa cells was assessed. A shRNA-mediated stable knockdown of the confirmed key components of mitochondrial Ca^2+^ uptake allowed us to separately investigate the contribution of mitochondrial Ca^2+^ buffering from that of mitochondrial depolarization to SOCE. We also took advantage of recent findings showing that UCP2 is involved in mitochondrial Ca^2+^ sequestration upon intracellular Ca^2+^ release, but not in the buffering of entering Ca^2+^ (Waldeck-Weiermair et al., 2010b; Waldeck-Weiermair et al., 2011), whereas MCU is generally involved in mitochondrial Ca^2+^ uptake regardless the source of Ca^2+^ (Waldeck-Weiermair et al., 2013; De Stefani et al., 2011). Accordingly, the use of MCU^KD^ and UCP2^KD^ cell models enabled us to determine whether SOCE activation and maintenance is facilitated by local mitochondrial buffering of Ca^2+^ entering by SOCE or mitochondrial sequestration of Ca^2+^ released from ER.

In accordance with recent reports (Waldeck-Weiermair et al., 2011; Waldeck-Weiermair et al., 2013), mitochondrial Ca^2+^ uptake upon cell treatment with an IP_3_-generating agonist was strongly reduced in HeLa cells with a stable knockdown of UCP2 (supplementary material Fig. S1B), whereas the lack of this protein did not affect mitochondrial uptake of entering Ca^2+^ (supplementary material Fig. S1C). Although it has been hypothesized that UCP2 and UCP3 are preferentially located within sites of mitochondrial Ca^2+^ uptake that face areas of ER Ca^2+^ release (Waldeck-Weiermair et al., 2010a), it is still unknown why the knockdown of these proteins exclusively diminishes mitochondrial uptake of Ca^2+^ that is mobilized from the ER. Interestingly, UCP2 depletion reduced mitochondrial Ca^2+^ uptake upon treatment of the cells with histamine to the same extent as a stable knockdown of MCU (supplementary material Fig. S1B), the proposed pore-forming subunit of a mitochondrial Ca^2+^ channel (De Stefani et al., 2011). These findings show that actually both UCP2 and MCU are necessary to accomplish IP_3_-mediated mitochondrial Ca^2+^ uptake in HeLa cells, thus pointing to a possible functional interrelation of these proteins, which requires further investigation. Notably, our recent study identified three biophysically distinct mitochondrial Ca^2+^ channel currents, of which only the most frequently occurring one depends on the presence of MCU (Bondarenko et al., 2013). Our data, however, do not only confirm that both UCP2 and MCU are essential for mitochondrial Ca^2+^ uptake, but demonstrate that the two distinct proteins influence SOCE regulation in a similar manner, due to their common involvement in mitochondrial Ca^2+^ uptake.

Interestingly, a knockdown of UCP2, which, in contrast to MCU, is not essential for mitochondrial sequestration of entering Ca^2+^ (Waldeck-Weiermair et al., 2013), mimicked the inhibitory effect of MCU knockdown on IP_3_-triggered STIM1 activation (Fig. 1) and, in turn, SOCE (Fig. 4). These findings indicate that mitochondrial Ca^2+^ buffering at the mouth of IP_3_ receptors is the major mechanism by which mitochondria contribute to SOCE activation under conditions of physiological stimulation. Accordingly, our findings partially confirm a model of the mechanism by which mitochondria regulate SOCE in immune cells, introduced by Anant Parekh before STIM1 and Orai1 were described (Gilabert et al., 2001). According to this study, energized mitochondria and, hence, increased mitochondrial Ca^2+^ buffering, augments Ca^2+^-release-activated Ca^2+^ current (I_CRAC_), the electrophysiological correlate of SOCE (Hoth and Penner, 1992). They concluded that local mitochondrial Ca^2+^ buffering at sites of ER Ca^2+^ release facilitates store depletion and, hence, significantly supports SOCE activation by IP_3_ (Gilabert et al., 2001). Several (whole-cell) patch-clamp studies in immune cells have indicated that Ca^2+^-chelating compounds, such as BAPTA or EGTA, need to be added to the pipette solution to evoke a pronounced I_CRAC_ (Zweifach and Lewis, 1995; Glitsch et al., 2002). However, under such conditions of artificially increased intracellular Ca^2+^-buffering capacity, the mitochondrial contribution is not relevant for SOCE/I_CRAC_ (Gilabert et al., 2001). In agreement with these findings, our data confirm that STIM1 oligomerization, which represents a key step in SOCE activation, becomes independent of MCU- and UCP2-mediated mitochondrial Ca^2+^ buffering upon histamine stimulation in cells loaded with BAPTA-AM (Fig. 3). When Ca^2+^ was mobilized by SERCA inhibition, which is known to efficiently deplete the store through Ca^2+^ leakage, STIM1 oligomerization remained largely unaffected by the knockdown of MCU or UCP2 (Fig. 2C), indicating that intact mitochondrial Ca^2+^ uptake is not required for SOCE activation under these conditions. SERCA inhibition does not promote the formation of high-concentration Ca^2+^ micro-domains (Waldeck-Weiermair et al., 2013; Giacomello et al., 2010), which are pivotal to activate the low-Ca^2+^-sensitive mitochondrial Ca^2+^-uptake pathway (Rizzuto et al., 1999). Studies using both chemical indicators (Collins et al., 2001) and genetically encoded Ca^2+^ probes (Malli et al., 2005) have revealed that SERCA inhibition evokes only small and delayed mitochondrial Ca^2+^ signals. Accordingly, we can assume that, due to the lack of an efficient activation of mitochondrial Ca^2+^ uptake under these conditions, the contribution of MCU and UCP2 to STIM1 oligomerization is irrelevant.

**Fig. 7.**
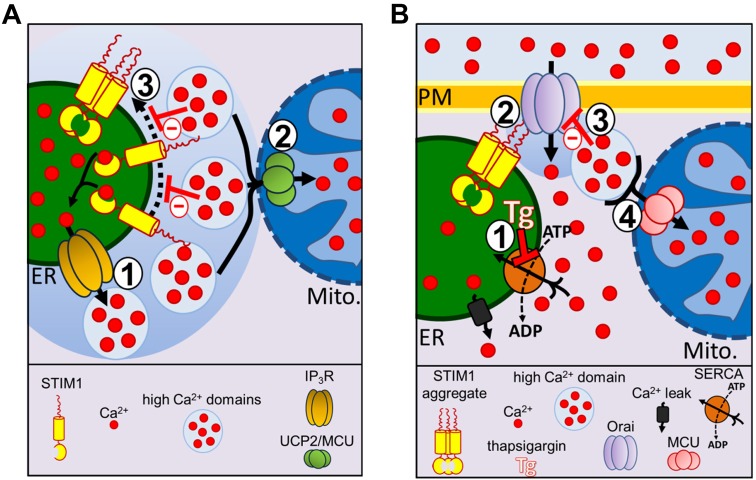
**Schematic showing the importance of mitochondrial Ca^2+^ uptake for STIM1 oligomerization and SOCE maintenance in HeLa cells.** (A) The impact of UCP2- and MCU-dependent mitochondrial Ca^2+^ uptake on STIM1 activation. Dashed arrow, STIM1 oligomerization; solid arrows, Ca^2+^ movements. (1) IP_3_-mediated Ca^2+^ release causes a reduction in the Ca^2+^ content of the ER, which triggers STIM1 oligomerization. However, local high-concentration Ca^2+^ micro-domains on the cytosolic side of the ER membrane restrain the formation of STIM1 oligomers. (2) Mitochondria (mito.) adjacent to sites of ER Ca^2+^ release act to sequester Ca^2+^ by a UCP2- and MCU-dependent pathway, which dissipates high-concentration Ca^2+^ micro-domains and, hence, facilitates STIM1 oligomerization. (3) Accordingly, STIM1 oligomerization upon IP_3_-mediated Ca^2+^ mobilization requires mitochondrial Ca^2+^ uptake, as Ca^2+^ buffering by the mitochondria results in both an efficient depletion of Ca^2+^ from the ER and, importantly, the dissipation of local cytosolic Ca^2+^ micro-domains at the ER surface. (B) MCU-dependent mitochondrial Ca^2+^ uptake counteracts Ca^2+^-dependent SOCE inactivation. Solid arrows, Ca^2+^ movements. (1) SERCA inhibition with thapsigargin induces strong ER Ca^2+^ depletion, leading to STIM1 oligomerization and punctae formation in superficial ER domains. (2) STIM1 aggregates interfere with plasma membrane (PM) Ca^2+^ channels such as Orai1, which leads to strong SOCE activation. (3) Upon Ca^2+^ addition to thapsigargin-treated cells, SOCE induces an increase in global and local cytosolic Ca^2+^ levels, and Ca^2+^ negatively impacts on SOCE. (4) MCU-dependent mitochondrial sequestration of entering Ca^2+^ is irrelevant for thapsigargin-induced SOCE activation but essential to maintain SOCE – probably by abrogating the (slow) Ca^2+^-dependent inactivation of this Ca^2+^ entry pathway.

Experiments using genetically encoded, organelle-targeted Ca^2+^ probes have revealed that the ER Ca^2+^ homeostasis is controlled by mitochondria in a rather complex manner. Upon IP_3_-mediated cell stimulation, mitochondria are able to recycle Ca^2+^ that has been released from intracellular stores (Arnaudeau et al., 2001) and shuttle entering Ca^2+^ to ER Ca^2+^ reuptake sites (Malli et al., 2005), thus facilitating Ca^2+^ refilling of the ER, which counteracts store depletion and, hence, SOCE activation. In this study, we used D1ER, an ER-targeted cameleon (Palmer et al., 2004), to investigate the impact of MCU and UCP2 knockdown on the Ca^2+^ homeostasis of the ER. Our data show that D1ER signals are only slightly affected in MCU^KD^ or UCP2^KD^ cells when Ca^2+^ is mobilized with histamine in the presence of extracellular Ca^2+^ (Fig. 1B), indicating that reduced mitochondrial Ca^2+^ uptake to some extent attenuates the kinetics of IP_3_-mediated ER Ca^2+^ depletion. This observation conflicts with the idea that mitochondria recycle released Ca^2+^ back to the ER (Arnaudeau et al., 2001), whereas it confirms the assumption that local mitochondrial Ca^2+^ buffering facilitates IP_3_-mediated ER Ca^2+^ depletion (Gilabert et al., 2001). Interestingly, we found no differences in the kinetics of ER Ca^2+^ depletion between the three cell lines when ER Ca^2+^ was mobilized with histamine in the absence of extracellular Ca^2+^ (Fig. 2B). However, STIM1 oligomerization was clearly delayed in MCU^KD^ and UCP2^KD^ cells under these conditions (Fig. 2A), raising the question of why a difference in STIM1 activation occurs despite the same rate of ER Ca^2+^ depletion. This discrepancy might depend on the different Ca^2+^-binding affinities of D1ER and the EF-hand motif of STIM1. *In vitro* calibration of D1ER revealed a dissociation constant (Kd) of 60 µM (Palmer et al., 2004), whereas the Kd of the Ca^2+^ probe was estimated to be ∼220 µM *in vivo* (Rudolf et al., 2006). The Kd of the EF-hand domain of STIM1 proteins to detect ER Ca^2+^ fluctuations has been calculated to be between 200 and 600 µM (Stathopulos et al., 2006). Based on these clear differences, it appears feasible that small changes in [Ca^2+^]_ER_ are sensed by STIM1, but not by D1ER. Alternatively, mitochondrial Ca^2+^ buffering facilitates the reduction of [Ca^2+^]_ER_ primarily within subcompartments of the ER that are sensed by STIM1, whereas the global ER Ca^2+^ concentration remains unaffected. Such specialized compartments of the ER have recently been described and named as the pre-cortical ER. These structures display thin ER tubules linked to microtubules and are enriched in STIM1 proteins (Shen et al., 2011a). In addition, cortical ER sections, which are also enriched in STIM1 proteins, have been observed close to the plasma membrane (Shen et al., 2011a). Interestingly, these thin ER compartments do not contain Ca^2+^-binding chaperones, indicating a different mechanism of Ca^2+^ homeostasis within the cortical ER compared with that of the bulk ER. Accordingly, it is tempting to speculate that mitochondria, which have been shown to be tethered to certain ER areas (de Brito and Scorrano, 2008), preferentially interact with those STIM1-enriched specialized ER compartments. In addition, our data unveiled a clear discrepancy between the kinetics of ER Ca^2+^ depletion and STIM1 oligomerization in response to BHQ (Fig. 3C). Under these conditions, STIM1 oligomerization was fast, despite slow ER Ca^2+^ depletion. We, and others, have suggested previously that STIM1 punctae formation and oligomerization is indeed under the control of cytosolic Ca^2+^ (Malli et al., 2008; Shen et al., 2011b). As slow ER Ca^2+^ depletion with a SERCA inhibitor does not generate high-concentration cytosolic Ca^2+^ micro-domains on the ER surface, STIM1 oligomerization under these conditions is independent of mitochondrial Ca^2+^ uptake. This finding indicates that the lack of cytosolic Ca^2+^ hot-spot formation upon SERCA inhibition promotes STIM1 activation. In accordance with these observations, our findings highlight that, upon ER Ca^2+^ mobilization with a physiological IP_3_-generating agonist, UCP2- and MCU-dependent mitochondrial Ca^2+^ buffering is essential for STIM1 oligomerization. Therefore, we conclude that mitochondrial Ca^2+^ buffering in the vicinity of the ER predominately shapes cytosolic Ca^2+^ micro-domains, thus facilitating STIM1 activation (Fig. 7A). However, our data demonstrate that, upon irreversible SERCA inhibition with thapsigargin, a sustained Ca^2+^ entry through the SOCE pathway requires MCU-dependent mitochondrial Ca^2+^ uptake (Fig. 6B; Fig. 7B). This is a striking finding in view of an earlier study that questioned the formation of high-concentration Ca^2+^ micro-domains on mitochondria upon SOCE in HeLa cells (Giacomello et al., 2010). Therefore, it is tempting to speculate that the MCU-dependent transfer of entering Ca^2+^ into mitochondria controls the maintenance of SOCE by a mechanism other than by shaping subplasmalemmal Ca^2+^ hot spots.

Recently, an MCU knockout mouse was generated (Pan et al., 2013). Interestingly, MCU depletion resulted in a rather inconspicuous phenotype, which is comparable with that of UCP2 or UCP3 knockout mice (Arsenijevic et al., 2000). The lack of MCU was, however, associated with reduced Ca^2+^-stimulated mitochondrial ATP generation and skeletal muscle work (Pan et al., 2013). Typical STIM1-related functions, such as T-cell activation and skeletal muscle physiology (Frischauf et al., 2008), have not been specifically examined in MCU knockout mice thus far. However, both the clear inability of mitochondria to respond to increased levels of Ca^2+^ with increased ATP generation in MCU knockout cells and our findings demonstrating the importance of MCU in SOCE activation and maintenance point to mitochondrial ATP as a potential regulator of SOCE. Indeed, it has been suggested that ATP facilitates SOCE by activating certain kinases (Lang et al., 2012) and/or increasing the local Ca^2+^-buffering capacity (Montalvo et al., 2006). Additional studies are necessary to entirely characterize the molecular mechanisms by which mitochondrial Ca^2+^ handling and organelle metabolism interfere with SOCE activation, maintenance and termination in different cell types.

Our study, however, reveals that, under physiological conditions of IP_3_-mediated Ca^2+^ mobilization, the transfer of Ca^2+^ from the ER into mitochondria is essential for efficient STIM1 oligomerization and SOCE activation. Notably, the mitochondria–SOCE interaction we describe herein might be a cell-type-specific phenomenon. However, considering the versatile functions of STIM1 (Soboloff et al., 2012), the identification of any molecular mechanism by which mitochondria regulate the activation of this protein under physiological conditions of cell stimulation will help to improve our understanding of other STIM1-dependent cell signaling events as well.

## MATERIALS AND METHODS

### Chemicals and buffer solutions

Cell culture materials were obtained from PAA laboratories (Pasching, Austria). FCCP, oligomycin A, histamine, BHQ and EGTA were purchased from Sigma Aldrich (Vienna, Austria). Thapsigargin and BAPTA-AM were from Abcam^®^ (London, UK). Prior to experiments, cells were washed and maintained for 20 minutes in a HEPES-buffered solution containing 138 mM NaCl, 5 mM KCl, 2 mM CaCl_2_, 1 mM MgCl_2_, 1 mM HEPES, 2.6 mM NaHCO_3_, 0.44 mM KH_2_PO_4_, 0.34 mM Na_2_HPO_4_, 10 mM d-glucose, 0.1% vitamins, 0.2% essential amino acids and 1% penicillin-streptomycin, the pH of which was adjusted to 7.4 with NaOH. During the experiments, cells were perfused with a Ca^2+^-containing buffer, which consisted of 145 mM NaCl, 5 mM KCl, 2 mM CaCl_2_, 1 mM MgCl_2_, 10 mM d-glucose and 10 mM HEPES, the pH of which was adjusted to 7.4 with NaOH. During experiments in which a Ca^2+^-free solution was applied to the cells, the CaCl_2_ was replaced with 1 mM EGTA.

### Stable knockdown cell lines

HeLa SilenciX^®^ knockdown cell lines were purchased from Tebu-bio (Le-Perray-en-Yvelines, France). We used HeLa cells stably expressing scrambled shRNA (control) or shRNA against MCU (MCU^KD^) (Bondarenko et al., 2013) or the UCP2 (UCP2^KD^).

### Cell culture and transfection

HeLa cells were grown in Dulbeccos's Modified Eagle Medium (Sigma Aldrich) containing 10% fetal bovine serum, 100 U/ml penicillin and 100 µg/ml streptomycin, and they were plated on 30-mm glass coverslips. At 60–80% confluence, cells were transfected with 1.5 µg (per 30-mm well) of plasmid DNA encoding the appropriate sensor/fluorophore using TransFast^TM^ transfection reagent at 3 µg/well (Promega, Madison, WI) in 1 ml of serum- and antibiotic-free medium. Cells were maintained in a humidified incubator (37°C, 5% CO_2_, 95% air) for 16–20 hours prior to changing the culture medium. All experiments were performed either 24 hours or 48 hours after transfection.

### Validation of shRNA-mediated stable knockdown of MCU and UCP2 using RT-PCR

RNA was isolated from SilenceX control, MCU^KD^ and UCP2^KD^ HeLa cells using a Total RNA isolation kit (Peqlab Biotechnologie, Erlangen, Germany). For reverse transcription, a High Capacity cDNA Reverse Transcription Kit from Applied Biosystems (Live Technologies Corporation, Vienna, Austria) was used. The analysis of the expression of MCU and UCP2 was performed by conventional PCR using GoTaq Green master mix (Promega) and by real-time (RT)-PCR using QuantiFast SYBR Green RT-PCR kit (Qiagen, Germantown, MD) on a LightCycler 480 (Roche Diagnostics Deutschland, Mannheim, Germany). RNA polymerase II (RPOL2) was used as the housekeeping control. Primers for RPOL2 and MCU were obtained from Invitrogen (Live Technologies Corporation, Vienna, Austria), and their sequences were as follows: RPOL2 forward, 5′-CATTGACTTGCGTTTCCACC-3′; RPOL2 reverse, 5′-ACATTTTGTGCAGAGTTGGC-3′; MCU forward, 5′-TTCCTGGCAGAATTTGGGAG-3′; and MCU reverse, 5′-AGAGATAGGCTTGAGTGTGAAC-3′. For human UCP2, the QuantiTect^®^Primer Assay (Qiagen, QT00014140) was used.

### Measurement of mitochondrial membrane potential

HeLa cells were loaded with 1 µM of the ratiometric dye JC-1 (Invitrogen) in culture medium at 37°C for 40 minutes, washed with PBS, dissociated by trypsinization, centrifuged at 262 ***g*** for 5 minutes and resuspended in the Ca^2+^-containing buffer described above. JC-1 fluorescence was detected using a fluorescence spectrophotometer (Hitachi F-4500, Hitachi, Inula, Austria). JC-1 was excited at 490 nm and 540 nm and emission was collected at 540 nm and 590 nm, respectively. The basal fluorescence ratio was normalized to the ratio after dissipation of mitochondrial membrane by treatment with 10 µM FCCP.

### Measurement of cellular oxygen consumption rate

Cells were plated in XF96 polystyrene cell culture microplates (Seahorse Bioscience) at a density of 30,000 cells per well. After an overnight incubation, cells were washed and preincubated for 30 minutes in unbuffered XF assay medium (Seahorse Bioscience) supplemented with 5.5 mM d-glucose and 1 mM sodium pyruvate at 37°C in a non-CO_2_ environment. Oxygen consumption rates were subsequently measured using an XF96 extracellular flux analyzer.

### Quantification of total cellular ATP content using HPLC

Separation of adenine nucleotides (neutralized supernatant) after cell lysis was performed on a Hypersil ODS column (5 µm, 250×4-mm inner diameter), using a L2200 autosampler, two L-2130 HTA pumps and a L2450 diode array detector (all from Hitachi), as described previously (Khan et al., 2012).

### Intra- and intermolecular FRET measurements

Dynamic changes in [Ca^2+^]_ER_ and [Ca^2+^]_mito_ were followed in cells expressing the ER- or mitochondria-targeted cameleons D1ER (Palmer et al., 2004) and 4mtD3cpv (Jean-Quartier et al., 2012), respectively. STIM1-oligomerization was monitored by measuring intermolecular FRET between CFP–STIM1 and YFP–STIM1 (Deak et al., 2013). Experiments were performed on an inverted microscope (Axio Observer.A1, Zeiss, Göttingen, Germany) equipped with a polychromator illumination system (VisiChrome, Visitron Systems, Puchheim, Germany) and a thermoelectric-cooled CCD camera (Photometrics CoolSNAP HQ, Visitron Systems). Cells were imaged with a 40× oil-immersion objective (Zeiss). Excitation of the fluorophores was at 440±10 nm (440AF21, Omega Optical, Brattleboro, VT), and emission was recorded at 480 and 535 nm using emission filters (480AF30 and 535AF26, Omega Optical) mounted on a Ludl filterwheel. Results of FRET measurements are shown as the ratio of (F_535_/F_480_)/R_0_ (where R_0_ is the basal ratio), to correct for photobleaching and/or photochromism, as described previously (Waldeck-Weiermair et al., 2012).

### Single-cell Ca^2+^ imaging using fura-2 and 4mtD1GO-Cam

Cytosolic Ca^2+^ signals of single cells were monitored using the classical fura-2 technique on a digital imaging system, as described previously (Deak et al., 2013). Briefly, prior to experiments, cells were incubated with 2 µM Fura-2/am (TEFLabs, Austin, TX) for 45 minutes and were alternately illuminated at 340 and 380 nm, while fluorescence emission was gathered at 510 nm. For simultaneous cytosolic and mitochondrial Ca^2+^ measurements, 4mtD1GO-Cam transfected HeLa cells loaded with Fura-2/am were used. Co-imaging of the different fluorophores was achieved with a digital wide-field imaging system, the Till iMIC (Till Photonics, Gräfelfing, Germany), using a 40× objective (Zeiss). Fura-2 and the 4mtD1GO-Cam were alternately excited at 340 nm or 380 nm and at 477 nm, respectively, with an ultra-fast switching monochromator, the Polychrome V (Till Photonics), equipped with an excitation filter (E500spuv) and a dichroic filter (495dcxru, Chroma Technology Corp., VT). Emitted light was simultaneously collected at 510 nm (Fura-2 and GFP of GO-Cam) and at 560 nm (FRET-channel of GO-Cam) using a single beam splitter design (Dichrotome, Till Photonics) that was equipped with a dual band emission filter (59004m ET Fitc/Tritc Dual Emitter) and a second dichroic filter (560dcxr, Chroma Technology Corp.). Images were recorded with a CCD camera (AVT Stringray F145B, Till Photonics). The digital imaging system was controlled by the live-acquisition software v2.0.0.12 (Till Photonics), as described previously (Waldeck-Weiermair et al., 2012).

### Mn^2+^ quench experiments

Fura-2/am-loaded HeLa cells were perfused with the Ca^2+^-containing experimental buffer supplemented with 100 µM MnCl_2_. Mn^2+^ quenching of cytosolic fura-2 fluorescence upon the addition of 100 µM histamine was measured by using the Ca^2+^ imaging system described above at an excitation wavelength of 360 nm.

### Confocal analysis and 3D rendering

High resolution *Z*-scan imaging of subcellular structures was performed in cells coexpressing D1ER and the mitochondria-targeted DsRed. Images were acquired with an array confocal laser scanning microscope, built on an inverse fully automatic microscope equipped with VoxCell Scan^®^ (VisiTech, Visitron Systems) and a 100× objective (Plan-Fluor 100×/1.45 oil, Zeiss). Fluorophores were illuminated at 488 nm (120 mW diode laser, Visitron Systems) and 515 nm (50 mW, VSLaserModul, Visitron Systems). Emitted light was acquired with a CCD camera (CoolSNAP-HQ, Photometrics,) using the emission filters ET535/30m and E570LPv2 (Chroma Technology) mounted on a computer-controlled fast-filter wheel (Ludl Electronic Products). All devices were controlled by VisiView Premier Acquisition software (Visitron Systems). The ER and mitochondria *z*-stacks were deconvoluted using the iterative quick maximum likelihood estimation algorithm (QMLE) of Huygens 2.4.1p3 (SVI, Hilversum, Netherlands). Subsequently, combined three-dimensional rendering of the organelles was performed with Imaris 3.3 software (Bitplane AG, Zurich, Switzerland). Quantitative mitochondrial shape analysis and colocalization computations were performed with the integrated morphometric analysis plug-in of MetaMorph 7.7.0.0 software (Visitron). The intensity threshold values of shape analysis and colocalization computations did not significantly differ within all the samples analyzed and were determined over a range that completely eliminated background fluorescence but preserved organelle structures.

### Statistics

Data shown represent the mean±s.e.m., where *n* reflects the number of cells. Statistical analyses were performed by using the unpaired Student's *t*-test, and *P*<0.05 was considered to be significant.

## Supplementary Material

Supplementary Material
