# Supplementary material for: IP3-mediated STIM1 oligomerization requires intact mitochondrial Ca2+ uptake
Source: J Cell Sci. 2014 Jul 1;127(13):2944–55. doi: 10.1242/jcs.149807 (PMC4077590; doi:10.1242/jcs.149807)
Supplement: Supplementary Material [file supp_127_13_2944__index.html]

IP3-mediated STIM1 oligomerization requires intact mitochondrial Ca2+ uptake — Supplementary Material 

# IP3-mediated STIM1 oligomerization requires intact mitochondrial Ca2+ uptake

## JCS149807 Supplementary Material

**Files in this Data Supplement:**

- **Supplementary Material**
