# Supplementary material for: IP3-mediated STIM1 oligomerization requires intact mitochondrial Ca2+ uptake
Source: J Cell Sci. 2014 Jul 1;127(13):2944–55. doi: 10.1242/jcs.149807 (PMC4077590; doi:10.1242/jcs.149807)
Supplement: Supplementary Material [file supp_127.13.2944_JCS149807.pdf]

Supplementary Material to:

Inositol-1,4,5-trisphosphate (IP_3)-mediated STIM1 oligomerization requires intact mitochondrial Ca^{2+} uptake

A. T. Deak[1], S. Blass[1], M. J. Khan[1], L. N. Groschner[1], M. Waldeck-Weiermair[1], S. Hallström[2], W. F. Graier[1], R. Malli[1]*

Affiliations:

[1]From the Institute of Molecular Biology and Biochemistry, Center of Molecular Medicine, Medical University of Graz, 8010-Graz, Austria.

[2]From the Institute of Physiological Chemistry, Center of Physiological Medicine, Medical University of Graz, 8010-Graz, Austria.

*To whom correspondence should be addressed: Roland Malli, Institute of Molecular Biology and Biochemistry, Molecular and Cellular Physiology Research Unit, Center of Molecular Medicine, Medical University of Graz, Harrachgasse 21/III, Graz, Austria. Phone: +43-316-380-7565; Fax: +43-316-380-9615; E-mail: roland.malli@medunigraz.at

Supplementary Material Content

Supplementary Figure Legends **(Figs. S1 and S2)**

Fig. S1. Characterization of $[Ca^{2+}]_{mito}$, metabolic status and organelle morphology in MCU^{KD} and $UCP2^{KD}$ cells

Fig. S2. Characterization of histamine-evoked cytosolic Ca^{2+} signals in MCU^{KD} and $UCP2^{KD}$ cells

Supplementary Figure Legends

Figure S1. Characterization of $[Ca^{2+}]$mito, the metabolic status, and the organelle morphology in MCU^{KD} and $UCP2^{KD}$ cells

(A) Knock-down efficiency of MCU and UCP2 was validated with qPCR in the SilenceX knock-down HeLa cell lines (n=9 for each samples). *p<0.05 vs. Control. **(B)** Mitochondrial Ca^{2+} signals upon stimulation with 100 µM histamine in the absence of extracellular Ca^{2+} were measured in Control (black, n=21), MCU^{KD} (red, n=19) and $UCP2^{KD}$ (blue, n=19) cells expressing 4mtD3CPV. Curves indicate mean ± s.e.m. *p<0.05 vs. Control. **(C)** Mitochondrial Ca^{2+} signals upon Ca^{2+} re-addition following 1 µM thapsigargin treatment in the absence of extracellular Ca^{2+} were measured in the same cells shown in (B). Curves indicate mean ± s.e.m. *p<0.05 vs. Control **(D)** Mitochondrial membrane potential was measured using the ratiometric dye JC-1 (n=3 for each cell line). The basal fluorescence ratio was normalized to the ratio after 10 µM FCCP addition**.** **(E)** The oxygen consumption rate in control (white column; n=30 wells), MCU^{KD} (red column; n=32 wells) and $UCP2^{KD}$ (blue column; n=30 wells) were measured using the Seahorse technology. **(F)** Columns represent the whole cellular ATP content of Control (white column, n=4), MCU^{KD} (red column, n=6) and $UCP2^{KD}$ (blue column, n=5) cells. The grey column represents the ATP content of control cells that were kept for 30 minutes in glucose-free medium (n=4). **(G)-(I)** Confocal analysis of mitochondrial and ER structures in HeLa cells expressing mtDsRed (red) and D1ER (green). **(G)** Images are representative for Control (top image), MCU^{KD} (middle image) and $UCP2^{KD}$ (bottom image). Scale bar is 10 µm. **(H)** Quantitative mitochondrial shape analysis based on shape factor values corresponding to mitochondrial morphology in Control (white columns, n=13), MCU^{KD} (red columns, n=14) and $UCP2^{KD}$ (blue columns, n=16) cells. Bar charts show distribution (mean ± s.e.m.) of mitochondrial shape factors calculated for all mitochondria within the middle plane of individual cells. **(I)** Co-localisation between ER and mitochondria in the different cell types (Control, MCU^{KD} and $UCP2^{KD}$) were defined as the percentage values of pixels that contain both fluorophores.

Figure S2. Characterization of histamine-evoked cytosolic Ca^{2+} signals in MCU^{KD} and $UCP2^{KD}$ cells

(A)-(C) Curves indicate single-cell cytosolic Ca^{2+} signals (thin curves) and their respective mean ± s.e.m. (thick curves) upon 100 µM histamine addition in fura-2/am loaded Control (left panels,) MCU^{KD} (left middle panels) and $UCP2^{KD}$ (right middle panels). Right panels summarize Ca^{2+} responses of different cells (mean ± s.e.m.). **(A)** Cell stimulation in a Ca^{2+}-free (EGTA) extracellular medium in Control/MCU^{KD}/$UCP2^{KD}$ cells (n= 76/76/65) **(B)** Cytosolic Ca^{2+} signals of Control/MCU^{KD}/$UCP2^{KD}$ cells (n=43/39/36) expressing Orai(E106Q)-YFP **(C)** Control/MCU^{KD}/$UCP2^{KD}$ cells were pretreated with FCCP/Oligomycin (2µM each) prior to stimulation (n=54/40/56)

Figure S1

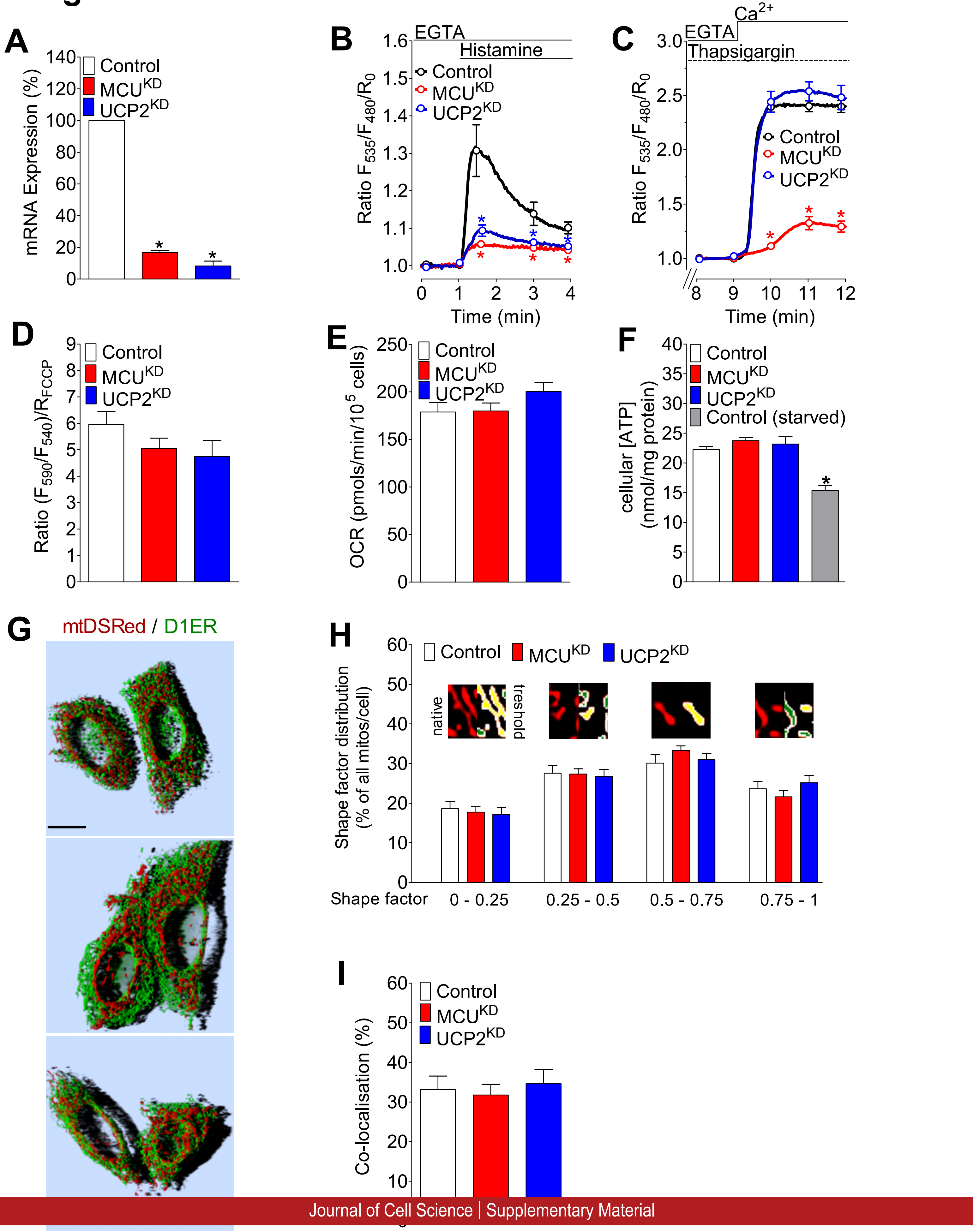
A
mRNA Expression (%)
Control
MCU^KD
UCP2^KD
B
EGTA
Histamine
Ratio F535/F480/R0
Time (min)
C
Ca2+
EGTA
Thapsigargin
Ratio F535/F480/R0
Time (min)
D
Ratio (F590/F540)/RFCCP
E
OCR (pmols/min/10^5 cells)
F
cellular [ATP] (nmol/mg protein)
Control (starved)
G
mtDSRed / D1ER
H
Shape factor distribution (% of all mitos/cell)
native
treshold
Shape factor
0 - 0.25
0.25 - 0.5
0.5 - 0.75
0.75 - 1
I
Co-localisation (%)

Figure S2

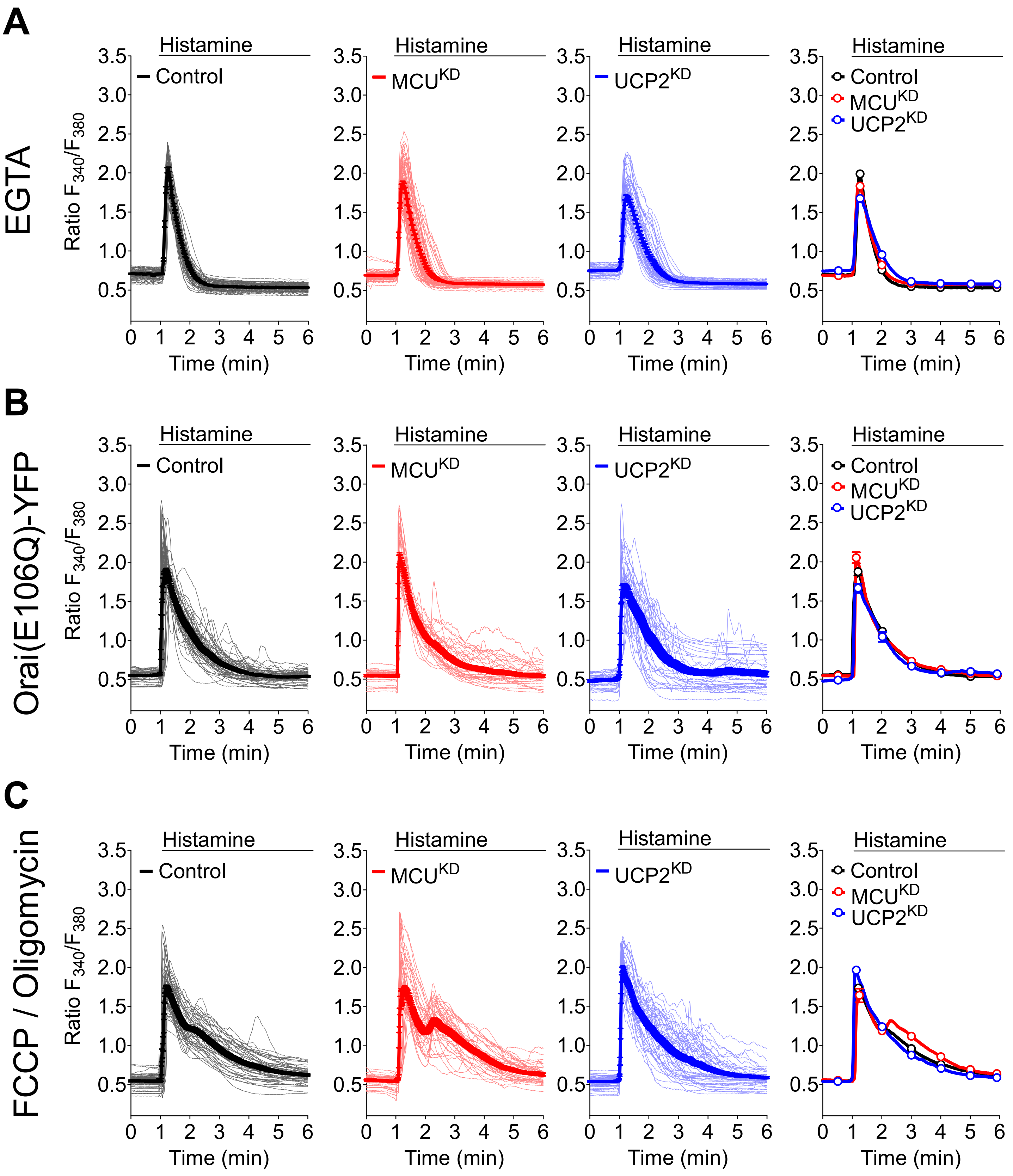
A
EGTA
B
Orai(E106Q)-YFP
C
FCCP / Oligomycin
Histamine
Control
MCU^{KD}
UCP2^{KD}
Ratio F_{340}/F_{380}
Time (min)
3.5
3.0
2.5
2.0
1.5
1.0
0.5
0
1
2
3
4
5
6
